# Supplementary material for: Dope Dyeing of Regenerated Cellulose Fibres with Leucoindigo as Base for Circularity of Denim
Source: Polymers (Basel). 2022 Dec 2;14(23):5280. doi: 10.3390/polym14235280 (PMC9738703; doi:10.3390/polym14235280)
Supplement: Supplementary file 1 [file polymers-14-05280-s001.zip › polymers-2050399-supplementary.pdf]

Supplementary material

Dope dyeing of regenerated cellulose fibres with leucoindigo as base for circularity of denim

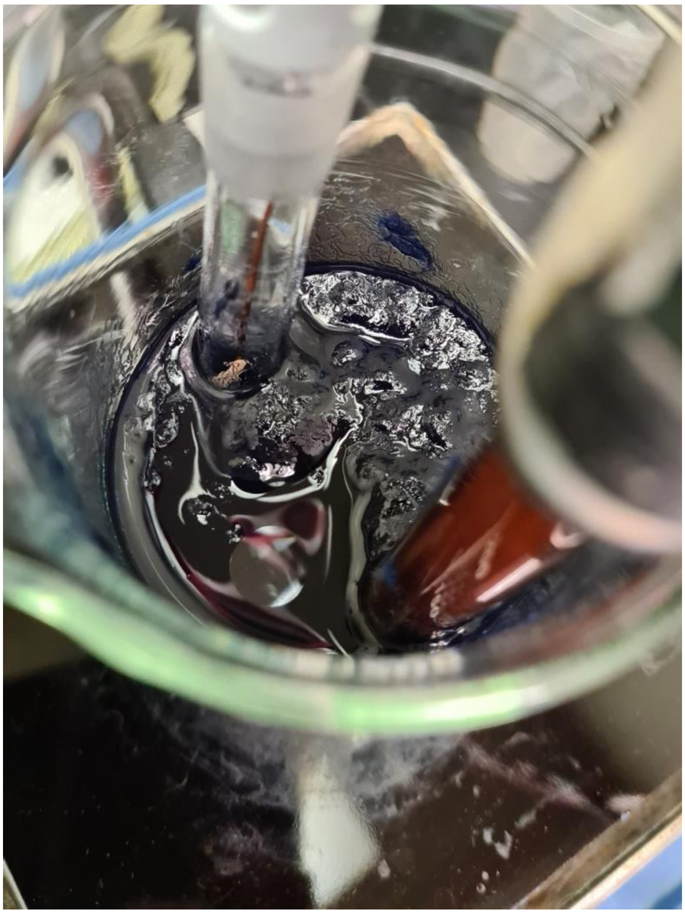

Figure S1. Steeping of indigo dyed CV in alkali (NaOH solution) before dissolution of xanthate.

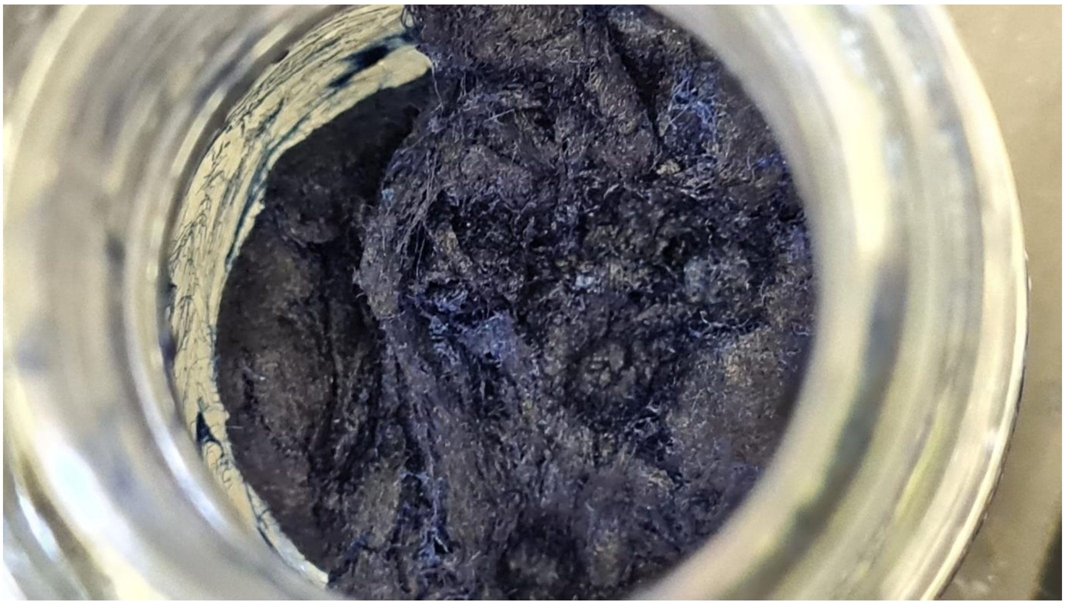

Figure S2. Alkalised indigo dyed CV fibres after xanthation.

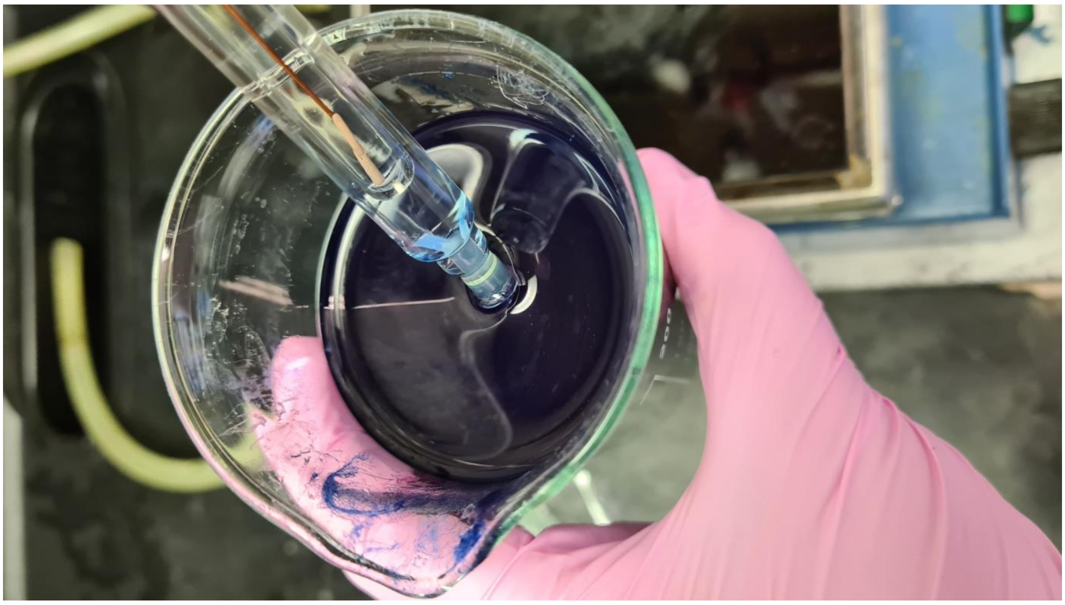

Figure S3. Completely dissolved xanthated indigo dyed CV after addition of alkali (NaOH solu-tion) before regeneration.

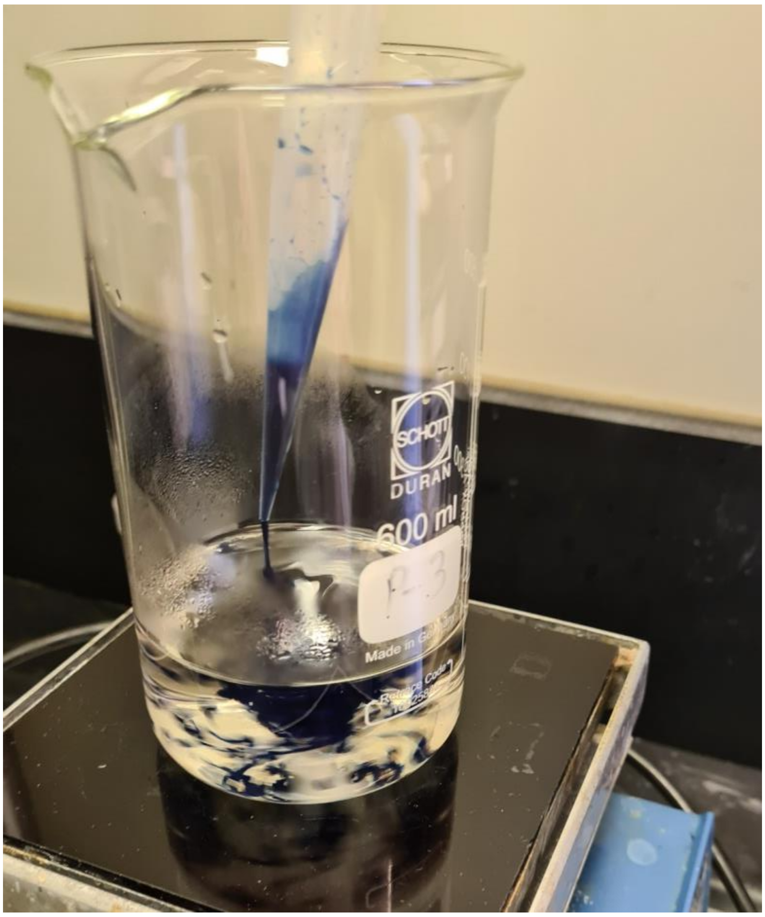

Figure S4. Regeneration of indigo dyed CV viscose solution by pouring it into diluted sulphuric acid.

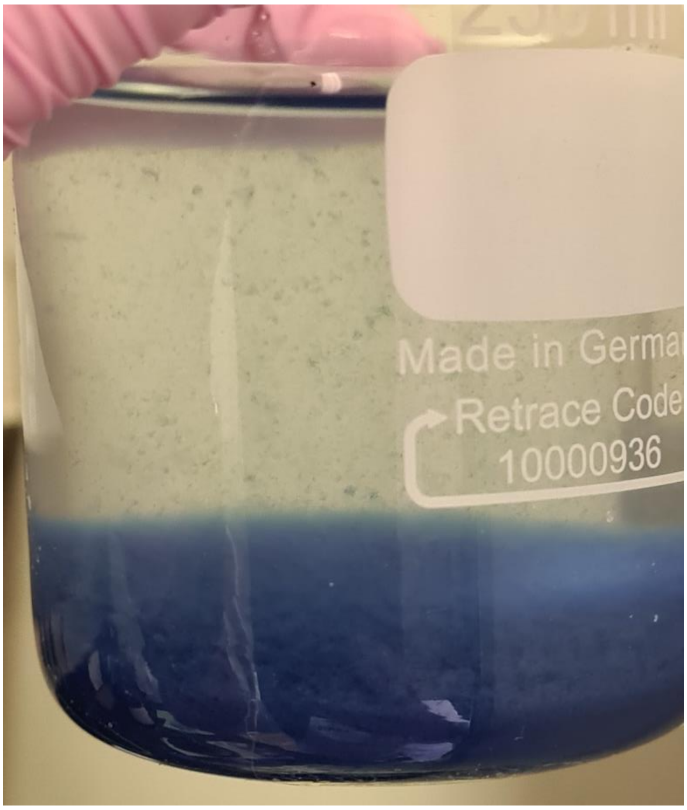

Figure S5. Regeneration bath after CV precipitation, some regenerated residues are settled to the bottom of the solution.

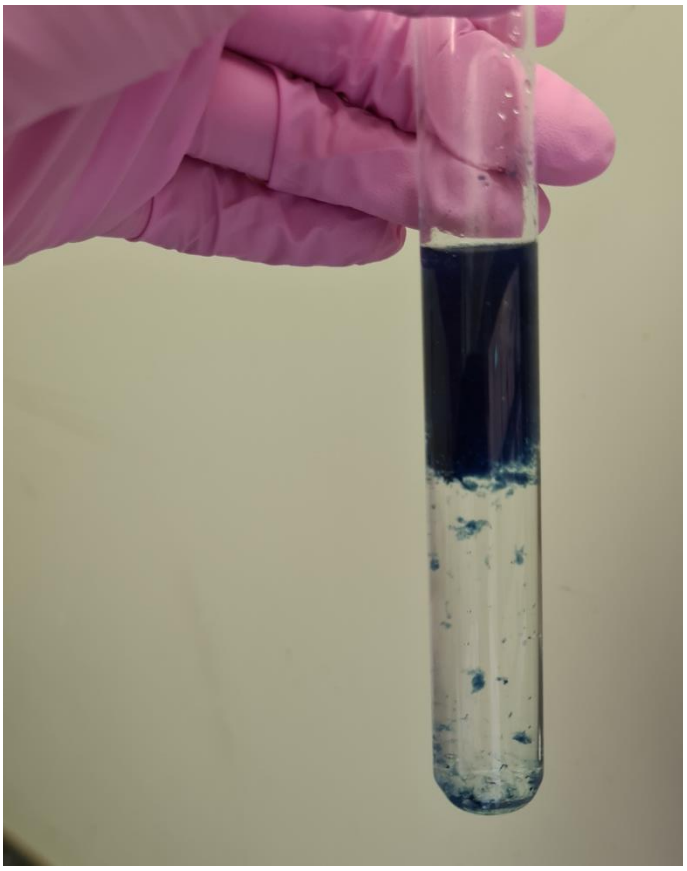

Figure S6. Separation of indigo containing regenerated cellulose from clear and uncoloured regeneration solution.

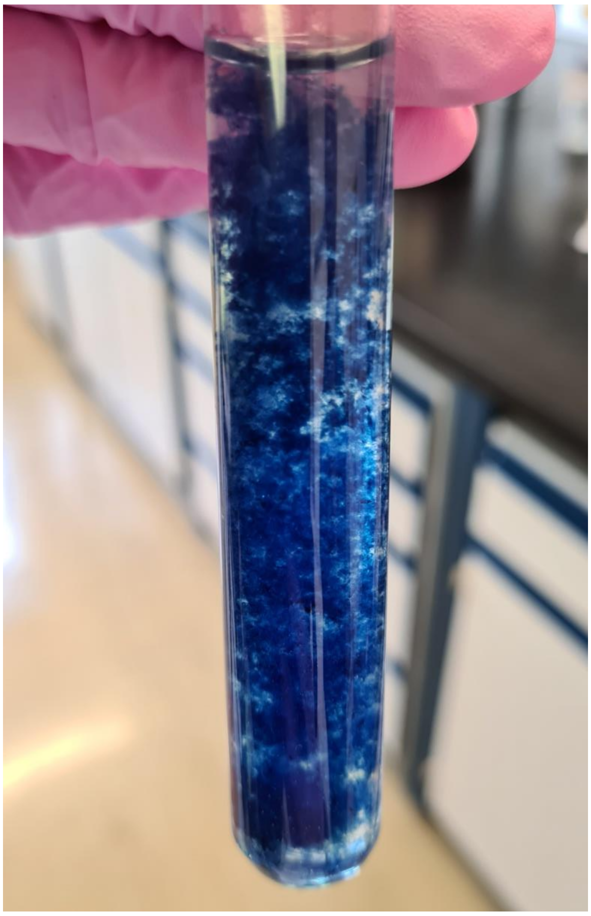

Figure S7. Regeneration bath and regenerated blue CV fibres.

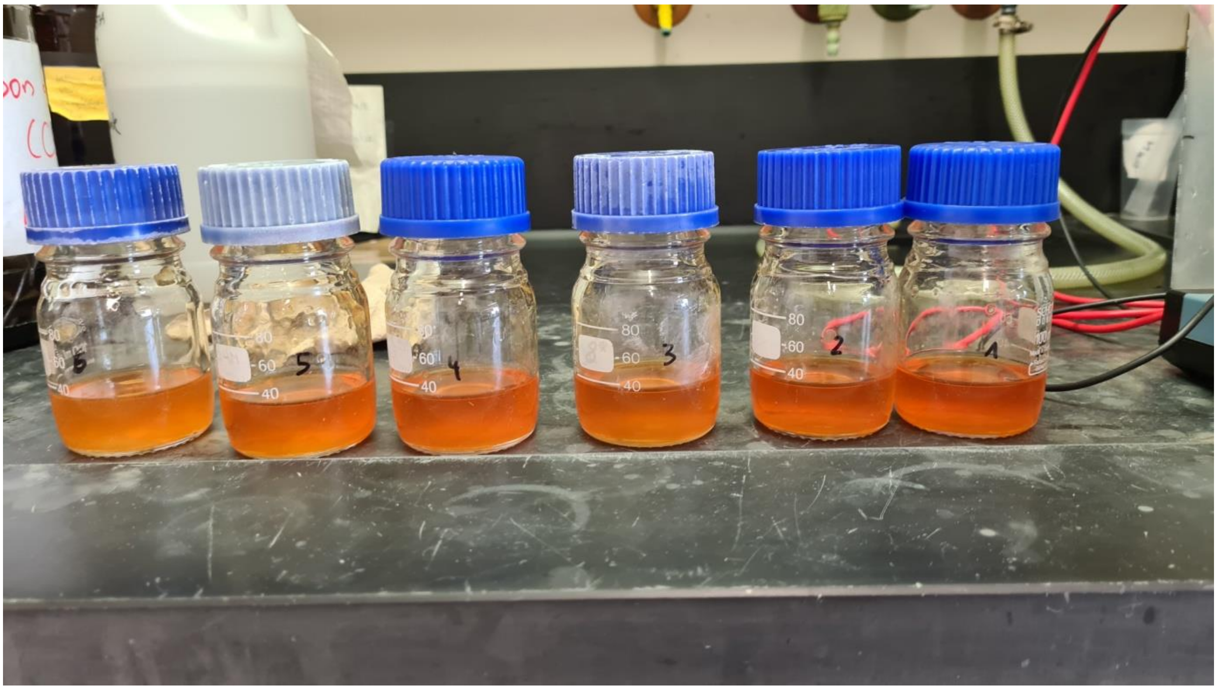

Figure S8. Dissolved xanthated viscose before addition of reduced indigo solution.

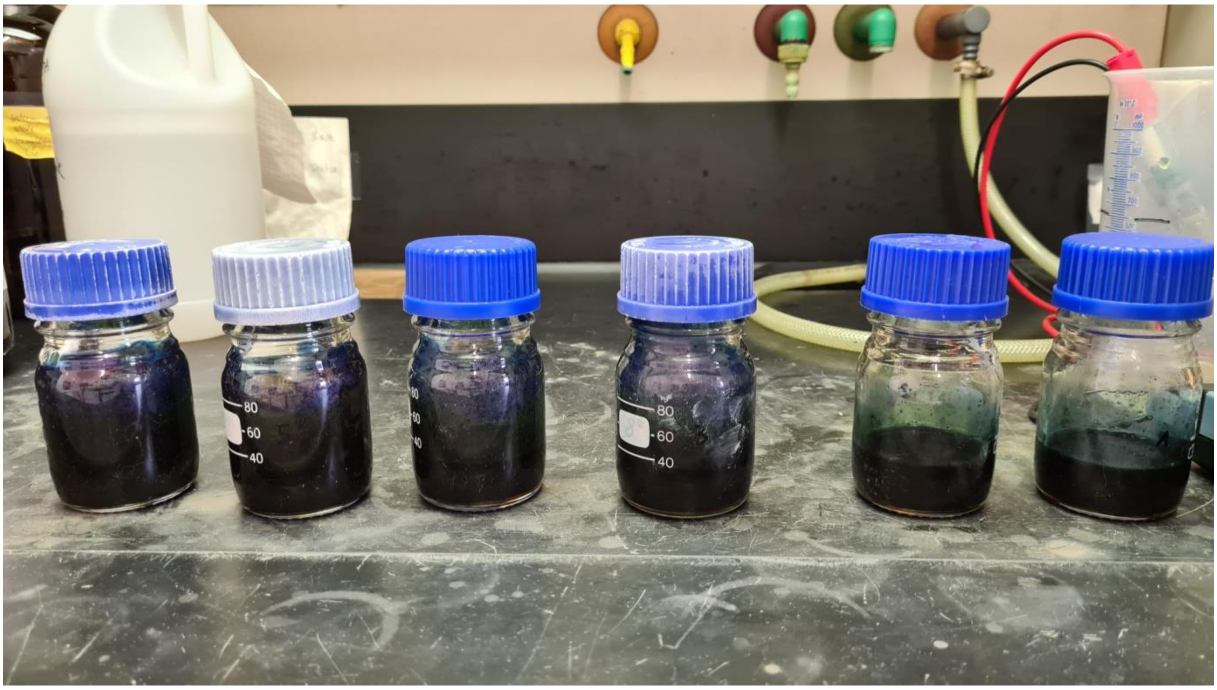

Figure S9. Dissolved xanthated viscose after addition of reduced indigo solution.

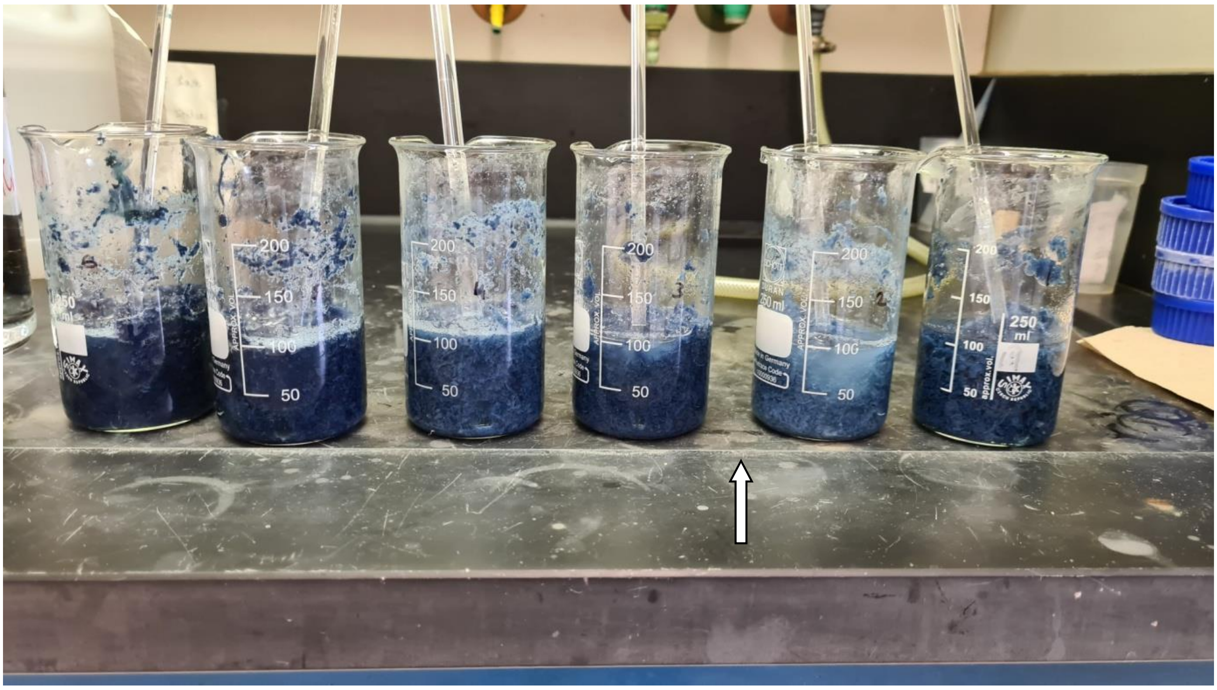

Figure S10. Cellulose regeneration in 10 %wt sulphuric acid (marked with arrow sample 2, regeneration bath is visible).

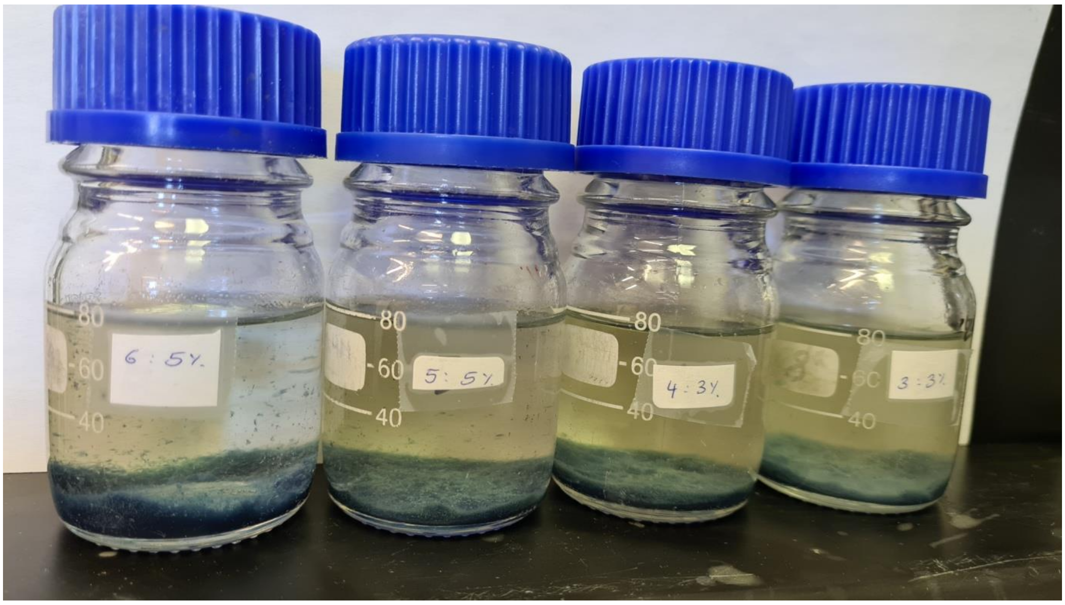

Figure S11. Collected regeneration baths after fibre regeneration, some reprecipitated CV settled to the bottom.

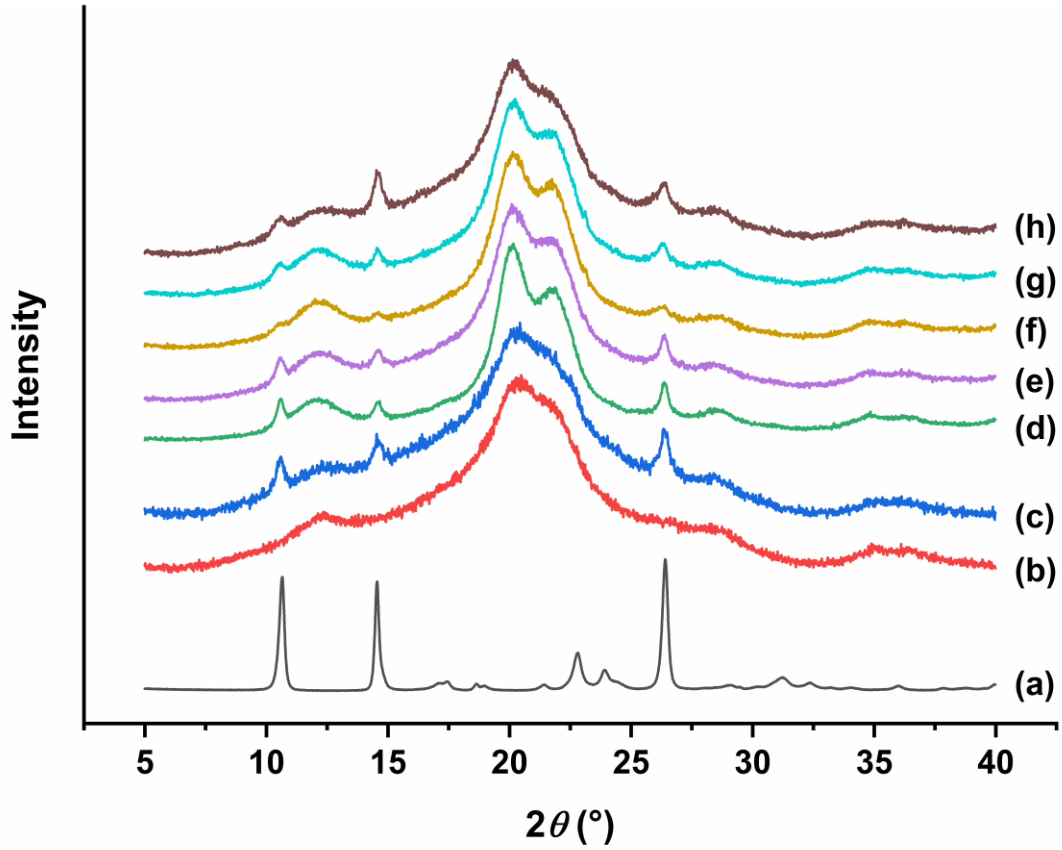

Figure S12. Representative XRD data. (a) indigo powder, (b) undyed cellulose fibres, (c) 3-dip indigo dyed CV (Table 2), (d) regeneration from H_2SO_4 + Na_2SO_4 of dyed fibres (sample G, Table 3), (e) 75% CS_2 xanthation of dyed fibres (sample C, Table 3), (f) addition of 0.81 %wt reduced indigo to spin dope (sample J, Table 4), (g) addition of 2.28 %wt reduced indigo to spin dope (sample K, Table 4), (h) addition of 4.19 %wt reduced indigo to spin dope (sample L, Table 4).
